# HNRNPA2B1 Affects the Prognosis of Esophageal Cancer by Regulating the miR-17-92 Cluster

**DOI:** 10.3389/fcell.2021.658642

**Published:** 2021-06-30

**Authors:** Kexin Li, Jiongyu Chen, Xiaoying Lou, Yiling Li, Benheng Qian, Danfei Xu, Yue Wu, Shaohui Ma, Donghong Zhang, Wei Cui

**Affiliations:** ^1^State Key Laboratory of Molecular Oncology, Department of Clinical Laboratory, National Cancer Center/National Clinical Research Center for Cancer/Cancer Hospital, Chinese Academy of Medical Sciences and Peking Union Medical College, Beijing, China; ^2^Central Laboratory, Cancer Hospital of Shantou University Medical College, Shantou, China; ^3^Department of Cardiology, The Second Affiliated Hospital of Wenzhou Medical University, Wenzhou, China

**Keywords:** esophageal cancer, m6A, miR-17-92 cluster, HNRNPA2B1, prognosis

## Abstract

N6-methyladenosine (m6A) is the most abundant RNA modification in eukaryotes. Accumulating evidence suggests that dysregulation of m6A modification significantly correlates with tumorigenesis and progression. In this study, we observed an increased expression and positive correlations of all 25 m6A regulators in esophageal cancer (ESCA) data obtained from the TCGA database. Through expression profiling of these regulators, a prognostic score model containing HNRNPA2B1, ALKBH5, and HNRNPG was established, and the high-risk subgroup exhibited strong positive correlations with ESCA progression and outcome. The risk score obtained from this model may represent an independent predictor of ESCA prognosis. Notably, the gene most frequently associated with increased risk was HNRNPA2B1; in ESCA, the increased expression of this gene alone predicted poor prognosis by affecting tumor-promoting signaling pathways through miR-17-92 cluster. An experimental study demonstrated that elevated HNRNPA2B1 expression was positively associated with distant metastasis and lymph node stage, and predicted the poor outcomes of ESCA patients. Knockdown of HNRNPA2B1 significantly decreased the expression of miR-17, miR-18a, miR-20a, miR-93, and miR-106b and inhibited the proliferation of ESCA cells. Therefore, our study indicated that the dynamic changes in 25 m6A regulators were associated with the clinical features and prognosis of patients with ESCA. Importantly, HNRNPA2B1 alone may affect the prognosis of patients with ESCA by regulating the miR-17-92 cluster.

## Introduction

Esophageal cancer (ESCA) is a common disease of the digestive system that is associated with poor prognosis and high mortality rates. As of 2015, the 5-year relative survival rate for ESCA was only 20% in the United States, and it is estimated that ESCA will be the sixth leading cause of cancer-related deaths in males by 2020 (Siegel et al., 2020). There are regional differences between Eastern and Western countries regarding the pathological types of ESCA. Esophageal squamous cell carcinoma (ESCC) is more prevalent in Southeast Asia, while 70–80% of all ESCAs are esophageal adenocarcinomas (EACs) in European and American countries (Arnold et al., 2015). Differences in living habits and genetic backgrounds are considered to be possible causes of these regional disparities. Unfortunately, due to the early occult clinical symptoms and lack of effective treatment, most patients with ESCA are diagnosed at an advanced stage and have poor outcomes (Alsop and Sharma, 2016). Therefore, obtaining a better understanding of the mechanisms underlying the occurrence and development of ESCA may facilitate a more effective treatment of patients with ESCA.

N6-methyladenosine (m6A) is the most prevalent and abundant RNA modification in eukaryotes (Motorin and Helm, 2011). The sequence motifs where m6A is detected are highly conserved, and this modification primarily occurs in the RRACH motif (R: adenine or cytosine; H: non-guanine). The functions of RNA metabolism are commonly regulated by methyltransferases (“writers”), demethylases (“erasers”), and RNA-binding proteins (“readers”). The m6A methylation is catalyzed by the methyltransferase complex including the core methyltransferase-like protein 3 (METTL3) and its adaptors (Bujnicki et al., 2002). The main m6A eraser acting on m6A is AlkB Homolog 5 (ALKBH5) and fat mass and obesity-associated protein (FTO) (Jia et al., 2011; Zheng et al., 2013). The “readers” included insulin-like growth factor 2 mRNA-binding proteins (IGF2BP1/2/3), YT521-B homology (YTH) family (YTHDF1/2/3 and YTHDC1/2), and heterogeneous nuclear ribonucleoproteins family members (HNRNPC/G/A2B1) (Dominissini et al., 2012; Schoenberg and Maquat, 2012; Fu et al., 2014; Wang et al., 2014; Huang et al., 2018). SRSF9, SRSF10, TRA2A, and EIF3A have also been reported to affect RNA metabolism depending on m6A modifications (Meyer et al., 2015; Xiao et al., 2016; Li et al., 2019; An et al., 2020). Previous research has shown that m6A mRNA modification is involved in physiological and pathological processes (Chen et al., 2018; Liu et al., 2018). Moreover, several studies have suggested that dysregulation of the m6A modification is significantly correlated with tumorigenesis and progression (Chen and Wong, 2020; Chen et al., 2020). Notably, the RNA-binding proteins play dominant roles in exerting the function of m6A RNA modification (Meyer and Jaffrey, 2017; Chen et al., 2019). For instance, the RNA-binding heterogeneous nuclear ribonucleoprotein A2/B1 (HNRNPA2B1) protein can bind m6A-containing sites and has an important effect on alternative splicing. Notably, HNRNPA2B1 could also mediate the m6A-dependent primary microRNA process and affect the production of miRNAs (Alarcón et al., 2015). However, the function of HNRNPA2B1 in miRNA regulation in ESCA has not been fully elucidated to date.

In the present study, we systematically analyzed the mRNA expression and interaction of 25 m6A regulators in ESCA data obtained from The Cancer Genome Atlas (TCGA) datasets. Next, we performed a molecular classification and established a prognostic score model with three m6A regulators. We further evaluated the correlation between m6A regulators and the clinical outcome and pathological characteristics. Next, we focused on the binding protein HNRNPA2B1 and investigated its expression and role in the prognosis of patients with ESCA. Moreover, we examined the mechanism by which HNRNPA2B1 may affect the prognosis of ESCA patients by regulating the miR-17-92 cluster.

## Manuscript Formatting

### Results

#### High Expression and Positive Correlation of 25 m6A Regulators in ESCA

**FIGURE 1:**
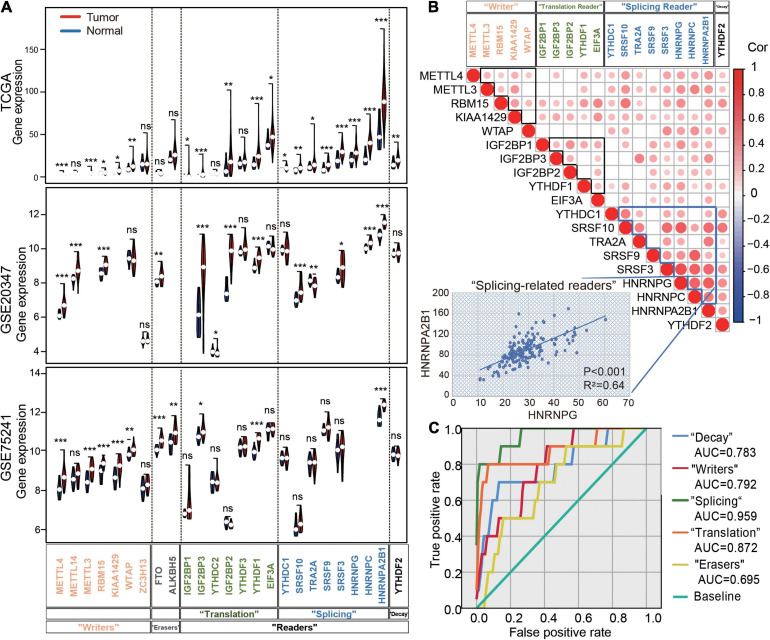
Expression and correlation of m6A regulators in ESCA. **(A)** Violin plots of m6A regulator expression in normal and tumor samples in ESCA from TCGA datasets, GSE20347 and GSE75241. **(B)** The correlation heatmap of the 16 m6A regulators in ESCA. The scatter plot shows the correlation between HNRNPG and HNRNPA2B1. **(C)** The receiver operating characteristic curve (ROC) curves for the prediction of normal and tumor samples using 25 m6A regulators in ESCA from TCGA datasets. AUC: the area under the curve. *P* values were calculated using the Wilcoxon rank-sum test (*P < 0.05; **P < 0.01; ***P < 0.001).

To understand the regulation of m6A regulators in ESCA, we first analyzed the expression profiling of 25 m6A regulators in 159 ESCA and 10 normal esophageal tissues based on the TCGA dataset as a test set. Interestingly, nearly all m6A regulators were significantly increased in ESCA tissues compared with normal samples (Figure 1A). In addition, we selected the two Gene Expression Omnibus (GEO) datasets as validation sets (GSE20347 and GSE75241). The results showed m6A regulators significantly increased in ESCA tissues compared with normal samples and HNRNPA2B1 presented the most abundant expression in esophageal tissues (Figure 1A). Next, we identified strong positive correlations among 19 regulators, especially for the “splicing readers” subgroup in all ESCA tissues. In particular, HNRNPA2B1 was significantly correlated with other “splicing readers,” such as HNRNPG (*R*^2^ = 0.64, *P* < 0.001) (Figure 1B). To explore the diagnostic value of each group in esophageal tissues, we performed the receiver operating characteristic (ROC) curve analysis and observed a higher area under the curve (AUC) in the “splicing readers,” “decay,” “writers,” “translation,” and “erasers” subgroups. Notably, splicing-related readers showed the highest diagnostic performance (AUC = 0.959) (Figure 1C). The results suggested dynamic changes in m6A regulators in ESCA development.

#### Molecular Classification by m6A Regulators in ESCA

Due to the current lack of clinical tumor molecular classification for ESCA, we investigated the role played by m6A regulators in molecular classification. Based on the expression data of m6A regulators, we classified 123 ESCA patients through a consensus clustering approach.

**FIGURE 2:**
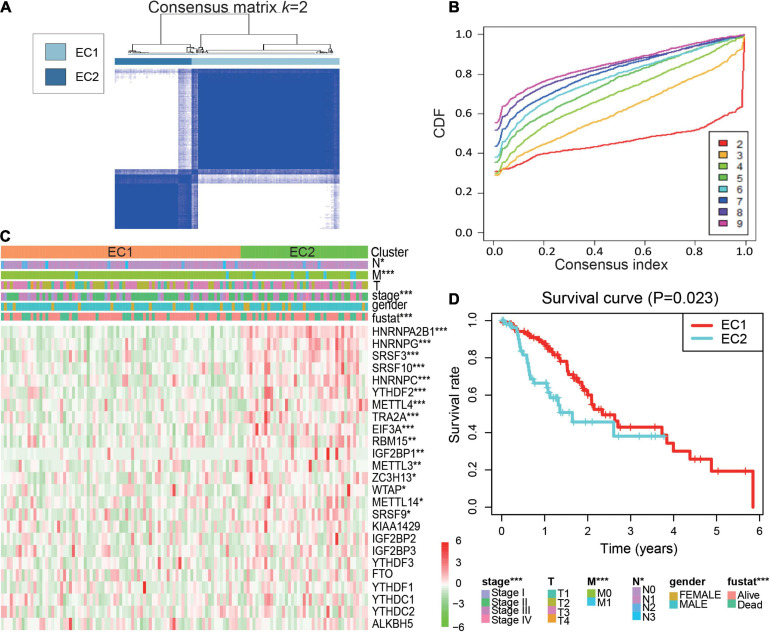
Molecular classification by 25 m6A regulators in ESCA. **(A)** Consensus clustering of cumulative distribution function (CDF) for *k* = 2; **(B)** Relative change in the area under the CDF curve for *k* = 2–9; **(C)** Heatmap and clinicopathological features of the two clusters defined by the consensus expression of the m6A regulators. EC1, esophageal cancer cluster 1; EC2, esophageal cancer cluster 2; **(D)** Kaplan–Meier overall survival (OS) curves of the ESCA patients. *P* values were calculated using *t*-test or Cox test (*p < 0.05; **p < 0.01; and ***p < 0.001).

In this instance, *k* = 2 was considered the best number of groups using the expression signature of 25 m6A regulators. Therefore, ESCA patients were divided into two clusters: 81 samples from the ESCA cluster 1 (EC1) and 42 samples from ESCA cluster 2 (EC2) subgroups (Figures 2A,B and Supplementary Figures 1A,B).

Next, the relationships of tumor subtype with the pathological characteristics and clinical outcome were analyzed. We found EC2 subgroup was remarkably correlated with advanced clinical stage, distant metastasis, lymph node metastases and overall survival (OS) of ESCA (Figure 2C and Supplementary Table 1). A Kaplan-Meier survival plot was generated to show the differences in outcomes between the two subgroups. We observed that the EC2 subgroup had a poorer prognosis than the EC1 subgroup (Figure 2D). Moreover, these results suggested that abundant m6A RNA regulators could affect the prognosis of patients with ESCA.

#### Prognostic Score Model Based on m6A Regulators in ESCA

**FIGURE 3:**
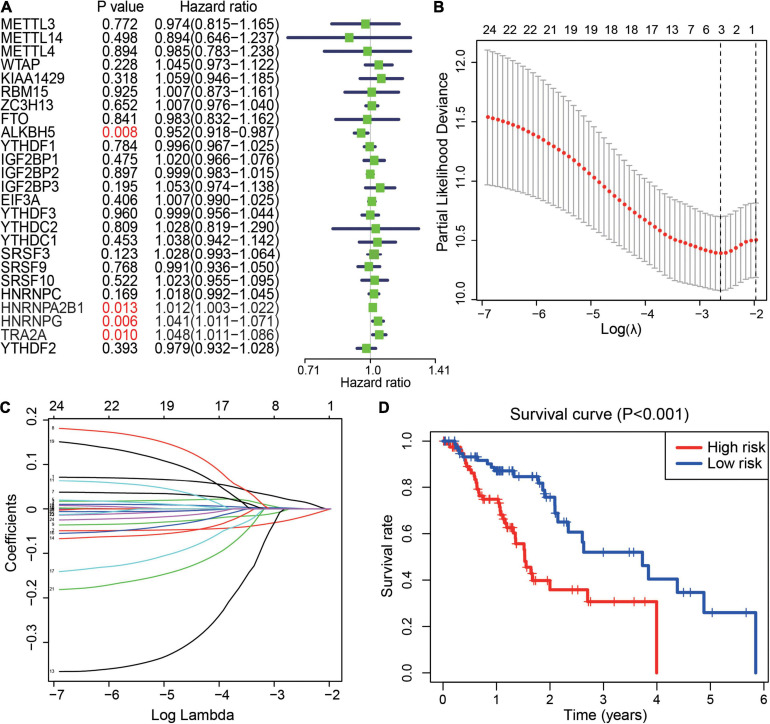
Prognostic value of m6A regulators and risk signature. **(A)** The distribution of the hazard ratios of 25 m6A regulators in ESCA. *P* value <0.05 displays in red. **(B,C)** Least absolute shrinkage and selection operator regression of the m6A regulators in ESCA. LASSO analysis to determine the optimal number of characteristic variables. **(D)** The prognostic score model for the prediction of the survival of ESCA patients in the “high-risk” subgroup and “low-risk” subgroup.

To further understand the prognostic value of m6A regulators in ESCA, a univariate Cox proportional hazards regression model was first performed to screen the potential genes initially. We observed that higher expression of ALKBH5 (HR = 0.952, 95% CI = 0.918–0.987) was associated with better survival in ESCA. Conversely, higher expression of three m6A splicing-related readers, HNRNPA2B1, HNRNPG, and TRA2A, was associated with worse survival in ESCA patients (Figure 3A). Next, the least absolute shrinkage and selection operator (LASSO) Cox regression algorithm was adopted to further screen the 25 genes in TCGA datasets. Consequently, ALKBH5, HNRNPA2B1, and HNRNPG were selected to construct the integrated prognostic score model, and the calculated parameters and coefficients were utilized to calculate the risk scores (Figures 3B,C). Moreover, 159 ESCA patients were divided into high-risk and low-risk groups based on the risk scores. We observed that the high-risk group had significantly poorer survival than the low-risk group in ESCA (Figure 3D). These results showed that m6A regulators played an important role in the prognosis of patients with ESCA.

**FIGURE 4:**
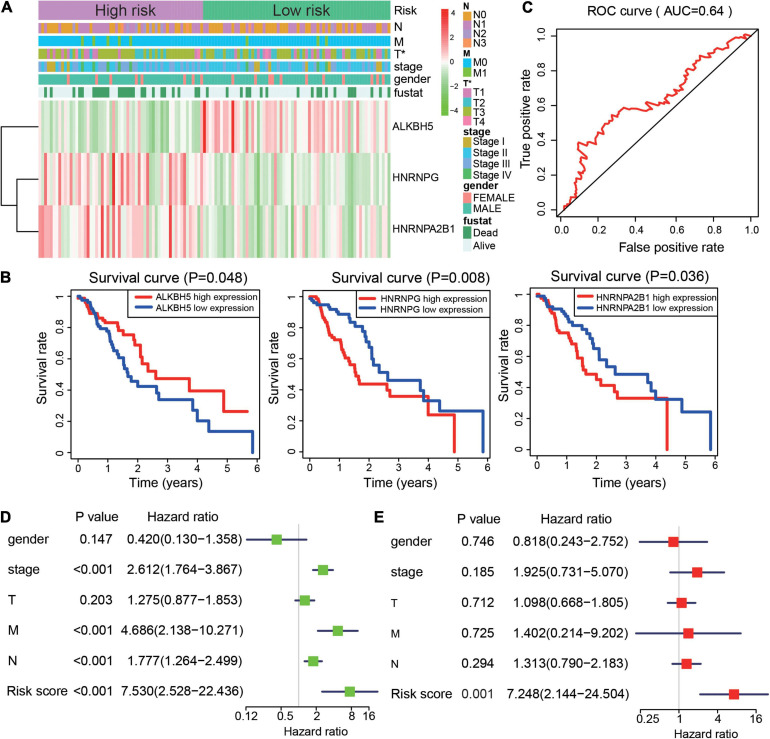
Risk signature is correlated with clinical features and outcome in ESCA. **(A)** Heatmap of ALKBH5, HNRNPG, and HNRNPA2B1 expression and clinical characteristics in the two subgroups of the ESCA cohort. **(B)** The prognostic value of ALKBH5, HNRNPA2B1, and HNRNPG expression in ESCA. **(C)** ROC curves showed the predictive efficiency of the risk signature. **(D,E)** Univariate **(D)** and multivariate **(E)** Cox regression analyses of the prognostic score model and clinical characteristics of ESCA. *P* value were calculated using Fisher’s exact test or Cox test (*P < 0.05).

Risk scores may provide prognostic value for clinical applications. We investigated the relationship between the risk scores and clinicopathological features of patients with ESCA. We found that the two groups had significant differences in tumor (T) volume. The high-risk group showed a larger tumor size (Figure 4A). Moreover, higher expression of ALKBH5 was present in the low-risk group, while HNRNPA2B1, and HNRNPG were more highly expressed in the high-risk group in ESCA (Figure 4A). After that step, we generated survival curves for ALKBH5, HNRNPA2B1, and HNRNPG in ESCA (Figure 4B). Next, risk scores were used to predict the 3-year survival rate of patients with ESCA by ROC (AUC = 0.64) curves to verify the effectiveness of the prognostic score model (Figure 4C). To elucidate the prognostic role played by risk scores in ESCA, we performed univariate and multivariate Cox regression analyses to identify whether the risk score could be used as an independent prognostic indicator. The results suggested that stage status, distant metastasis, lymph node metastasis and risk score were associated with OS in ESCA. More importantly, the risk score could be an independent indicator to predict the prognosis of patients with ESCA (Figures 4D,E). Taken together, these results suggested that the prognostic score model based on HNRNPA2B1, HNRNPG, and ALKBH5 could predict the outcome of patients with ESCA.

#### HNRNPA2B1 Can Participate in Tumor Progression by Regulating miR-17-92 Cluster Expression in ESCA

In the previous study, we found HNRNPA2B1 showed the highest expression among 25 m6A regulators in ESCA. The relative expression of 25 m6A regulators were compared in 13 types of common cancer cell lines (Supplementary Figure 2). Interestingly, we found that the HNRNPA2B1 mRNA was the highest expression in overall of 13-type of cancer cell lines. Similar finding was found in 33 kinds of clinical pan-cancer from TCGA database (Supplementary Figure 3). Together, HNRNPA2B1 might play an important role in tumor progression.

**FIGURE 5:**
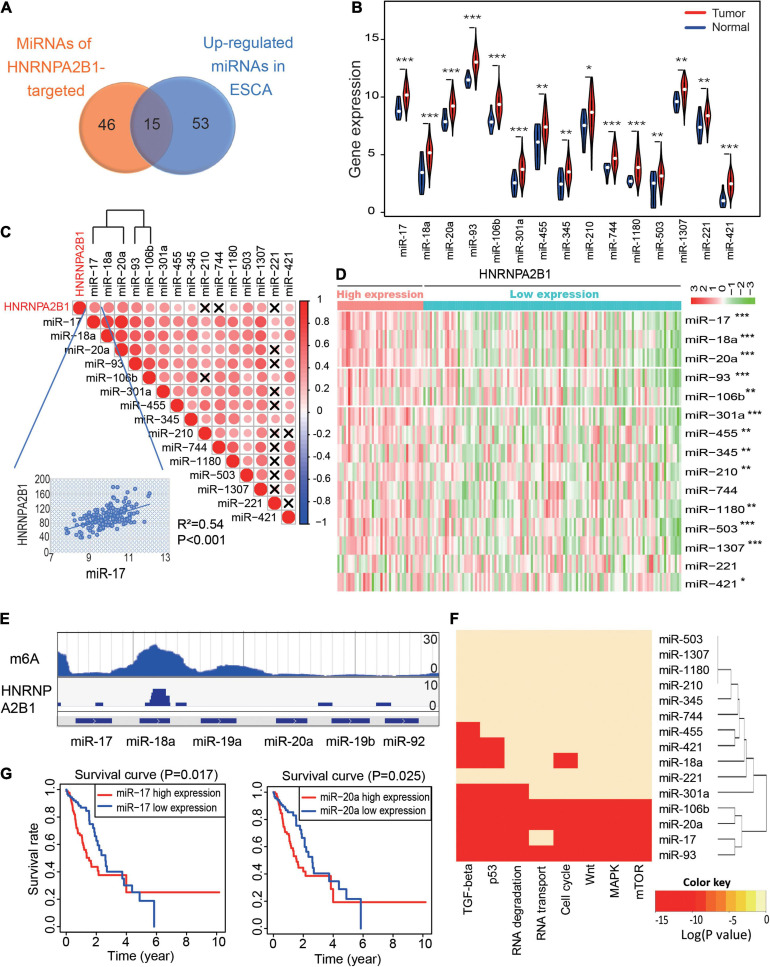
MiR-17-92 cluster expression positively correlates with HNRNPA2B1 expression and is associated with tumor progression in ESCA. **(A)** The Venn diagram of 15 microRNAs of the GEO datasets (GSE70061) and The Cancer Genome Atlas (TCGA) datasets. **(B)** Violin plots of the expression of the 15 miRNAs in normal and tumor samples in ESCA. **(C)** The correlation heatmap of the 15 miRNAs and HNRNPA2B1 expression in ESCA. ×, *P* > 0.05. The scatter plot shows the correlation between HNRNPA2B1 and miR-17. **(D)** Heatmap of the expression of 15 miRNAs between the high and low HNRNPA2B1 groups in ESCA. **(E)** The enrichment peak of m6A and HNRNPA2B1 in miR-17-92 cluster sites from the HITS CLIP sequence. **(F)** Gene ontology analysis of the 15 miRNAs. **(G)** Kaplan-Meier survival plots of patients grouped by the expression of miR-17 and miR-20a in ESCA. *P* values were calculated using Fisher’s exact test or Cox test (*P < 0.05; **P < 0.01; ***P < 0.001).

Recently, Alarcón et al. (2015) reported that HNRNPA2B1 can affect the production of miRNAs by mediating primary microRNA processing. Hence, we hypothesized that HNRNPA2B1 may be involved in the progression of ESCA by regulating miRNA expression. To verify this assumption, we collected data (GSE70061) from GEO datasets and found that 61 miRNAs were affected by HNRNPA2B1 depletion (Alarcón et al., 2015). Next, we observed that 68 miRNAs were significantly more highly expressed in ESCA than in normal tissues from TCGA datasets. Notably, 15 miRNAs were overlapped by increasing and targeting HNRNPA2B1 in ESCA (Figures 5A,B). Next, we examined the correlation between HNRNPA2B1 and 15 miRNAs and found that the expression of HNRNPA2B1 was positively associated with most of the 15 miRNAs in ESCA (Figure 5C). We also found that the HNRNPA2B1^*high*^ group presented significantly higher expression of almost all miRNAs in patients with ESCA in TCGA datasets (Figure 5D).

To address if HNRNPA2B1 regulates miRNA expression by m6A modification, we first re-analyzed the m6A-RIP-seq and HNRNPA2B1 HITS-CLIP-seq from GSE70061. The co-localization of HNRNPA2B1 and m6A modification was found on the miR-17-92 cluster (Figure 5E). Hence, the results indicated that HNRNPA2B1 might regulate the miR-17-92 cluster which dependents on m6A sites. Furthermore, a previous study reported that the miR-17-92 cluster played a critical role in the progression and development of ESCA (Chen et al., 2011). Consistently, our Gene Ontology (GO) analysis indicated that miR-17, miR-20a, miR-18a, miR-93, and miR-106b were enriched in several critical cancer-related signaling pathways, including the TGF-beta, p53, Wnt, MAPK, and mTOR signaling pathways, and these miRNAs were correlated with the cell cycle and RNA transport signaling pathways (Figure 5F). We also showed the important target genes for these miRNAs in Supplementary Table 2. Moreover, we found that higher expression of miR-17 and miR-20a was associated with poorer prognosis in patients with ESCA (Figure 5G). Taken together, these results suggested that HNRNPA2B1 might affect tumor-promoting signaling pathways by regulating the expression of the miR-17-92 cluster, leading to poor prognosis of ESCA patients.

#### High Expression of HNRNPA2B1 Is Associated With Poor Survival in ESCA

**FIGURE 6:**
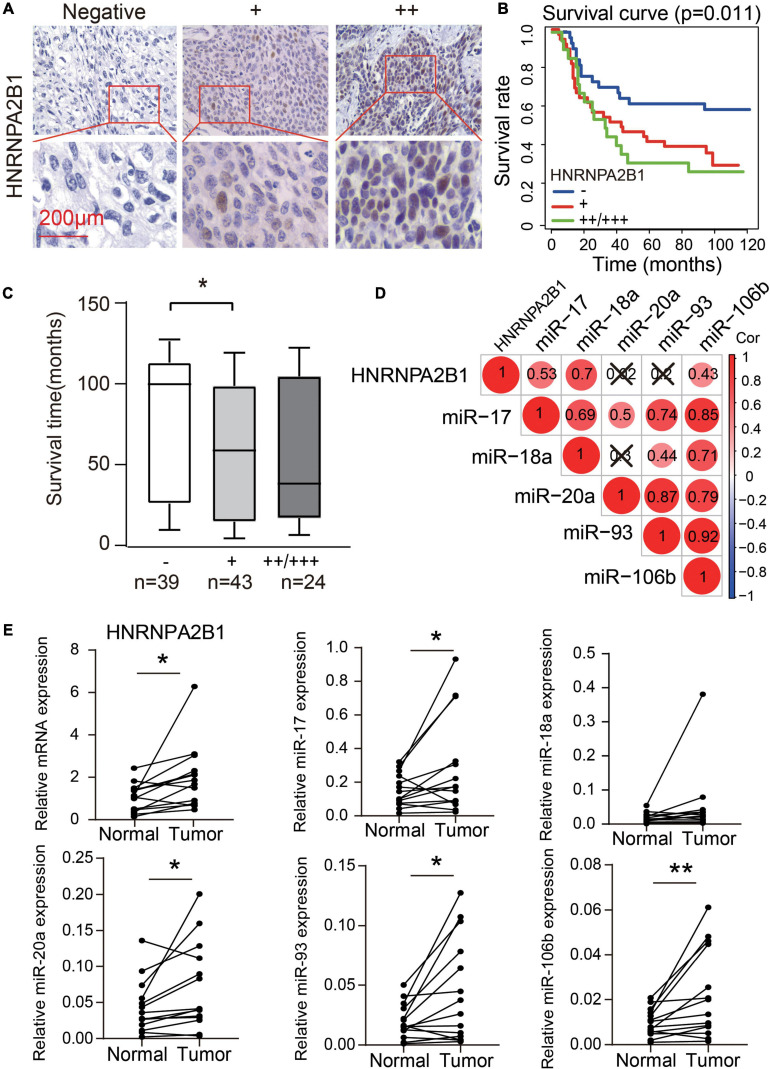
High expression of HNRNPA2B1 predicted poor survival in ESCA. **(A)** Representative HNRNPA2B1-negative and HNRNPA2B1-positive immunochemistry staining in ESCA tissues. **(B)** Kaplan-Meier analysis of overall survival in patients with or without HNRNPA2B1 expression in 106 clinical ESCA patients. **(C)** Box plot of overall survival time for patients with and without HNRNPA2B1 expression. **(D)** The correlation heatmap of the HNRNPA2B1 and 5 miRNAs in 14 paired ESCA tissues. ×, *P* > 0.05. **(E)** The differential expression of HNRNPA2B1 and 5 miRNAs in 14 paired ESCA tissues. *P* values were calculated using paired *t*-test (*P < 0.05 and **P < 0.01).

According to the results of bioinformatic analyses of m6A regulators, especially HNRNPA2B1, in ESCA, we assessed whether the expression of HNRNPA2B1 was associated with the progression of 106 cases of ESCA. We observed positive immunostaining for HNRNPA2B1 in the cell nucleus of 67/106 (63.21%) ESCA tissues (Figure 6A and Table 1). HNRNPA2B1 expression was positively associated with distant metastasis (χ^2^ = 8.352, *P* = 0.039) and lymph node stage (χ^2^ = 12.705, *P* = 0.048) in ESCA patients. However, no relationship was found with age, sex, tumor stage, or clinical stage (Table 1). On Kaplan-Meier survival analysis, ESCA patients with high HNRNPA2B1 protein had a worse outcome than those with low expression (*P* = 0.011, Figure 6B). In addition, the mean survival was shorter for patients with positive expression of HNRNPA2B1 (Figure 6C). Therefore, the ESCA patients with high expression of HNRNPA2B1 exhibited poor outcomes, and HNRNPA2B1 could serve as a prognostic predictor in ESCA.

**TABLE 1:** Association between HNRNPA2B1 expression and clinic pathological factors.

			**The expression of HNRNPA2B1**					
		***n***	**Negative**	**Positive**			**χ2**	***P* value**
			**−**	**+**	**++**	**+++**		
Gender	Male	73	26	27	18	2	3.615	0.306
	Female	33	13	16	3	1		
Age (years)	≤60	48	21	18	7	2	3.121	0.373
	>60	58	18	25	14	1		
Distant metastasis	Yes	4	1	0	3	0	8.352	0.039
	No	102	38	43	18	3		
Tumor Stage	T1	6	1	3	2	0	6.813	0.657
	T2	12	7	3	2	0		
	T3	58	21	26	10	1		
	T4	30	10	11	7	2		
Nodal Stage	N1	41	15	16	10	0	12.705	0.048
	N2	44	12	23	8	1		
	N3	21	12	4	3	2		
Clinical Stage	II	12	5	4	3	0	9.472	0.149
	III	90	33	39	15	3		
	IV	4	1	0	3	0		

We also collected the 14 paired esophagus cancer tissues and tumor-adjacent tissues to validate the bioinformatic analysis in TCGA datasets. We found that the significantly positive correlation between HNRNPA2B1 and miR-17/miR-18a/miR-106b in 14 paired esophagus cancer tissues and tumor-adjacent tissues (Figure 6D). Moreover, HNRNPA2B1, miR-17, miR-20a, miR-93, and miR-106b showed the higher expression in tumor tissues than tumor-adjacent tissues (Figure 6E). Our new results were consistent with the bioinformatic analysis from TCGA datasets.

#### Knockdown of HNRNPA2B1 Can Decrease the Expression of miRNAs and Inhibit Cell Proliferation in ESCA Cell Lines

**FIGURE 7:**
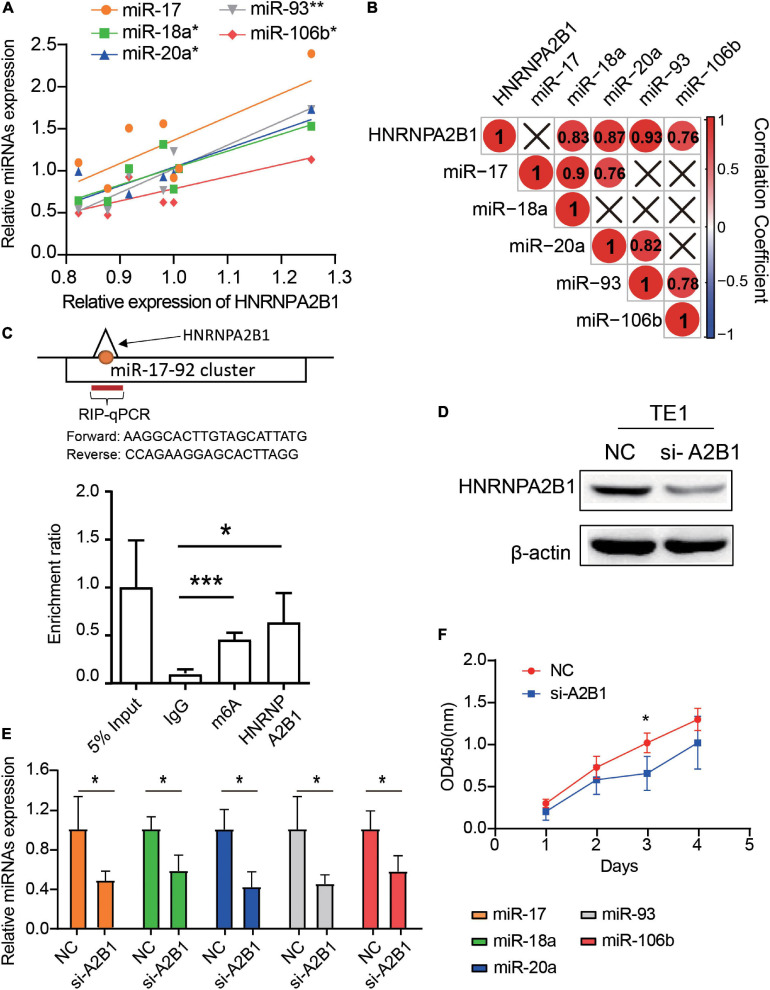
Knockdown of HNRNPA2B1 can downregulate the expression of miRNAs and suppress proliferation in ESCA cell lines. **(A)** The scatter plots show the correlation of HNRNPA2B1 and 5 miRNAs expression among 7 esophageal carcinoma cell lines through qRT-PCR. **(B)** The correlation heatmap of HNRNPA2B1 and 5 miRNAs in 7 ESCA cell lines. ×, *P* > 0.05. **(C)** RIP-qPCR analysis of the enrichment of miRNA on m6A and HNRNPA2B1 in TE1 cells. **(D)** HNRNPA2B1 expression was successfully knocked down by siRNA-HNRNPA2B1 transfection for 24 h in TE1 cells (*n* = 3). **(E)** MiRNA expression in TE1 cells with or without HNRNPA2B1 knockdown was measured by qRT-PCR. **(F)** Proliferation of TE1 cells with or without HNRNPA2B1 knockdown was measured by CCK-8 assays. *P* values were calculated using the Pearson correlation coefficient or Wilcoxon rank-sum test (*P < 0.05; **P < 0.01; and ***P < 0.001).

Based on the role played by HNRNPA2B1 in miR-17-92 cluster regulation, we investigated the correlation between HNRNPA2B1 and miR-17, miR-18a, miR-20a, miR-93 and miR-106b in seven ESCA cell lines. We found that the expression of HNRNPA2B1 had a significant positive correlation with the levels of miR-18a (*R*^2^ = 0.69, *P* = 0.021), miR-20a (*R*^2^ = 0.76, *P* = 0.0106), miR-93 (*R*^2^ = 0.87, *P* = 0.002), and miR-106b (*R*^2^ = 0.57, *P* = 0.049) (Figures 7A,B). To text if HNRNPA2B1 regulating miR-17-92 cluster by a m6A dependent pathway, we performed RIP and assessed if the HNRNPA2B1 and m6A could marked miRNAs. As shown in Figure 7C, m6A and HNRNPA2B1 could co-immunoprecipitate on the miR-17-92 cluster in TE1, which is consistent with our predicated result (Figure 5E). Next, we established transient HNRNPA2B1 knockdown models in TE1 cells with a small interfering RNA (siRNA) sequence. Successful knockdown of HNRNPA2B1 was confirmed at the protein level (Figure 7D). Knockdown of HNRNPA2B1 significantly downregulated the expression of miRNAs (Figure 7E). Moreover, knockdown of HNRNPA2B1 notably inhibited ESCA cell proliferation (Figure 7F). These data showed that HNRNPA2B1 affected the expression levels of miR-17-92 cluster and cell proliferation in ESCA.

## Discussion

In the present study, we demonstrated the generally high expression and positive correlation of 25 m6A regulators in ESCA. Next, we used 25 m6A regulators to classify the ESCA patients into two subgroups (EC1 and EC2) and established a prognostic score model (high-risk and low-risk) according to HNRNPA2B1, ALKBH5, and HNRNPG expression levels. These patterns exhibited a strong correlation with the clinical features and outcome of patients with ESCA. The risk score obtained from this model may represent an independent prognostic predictor for ESCA. Importantly, high expression of HNRNPA2B1 suggested a poor outcome in patients with ESCA and was positively associated with distant metastasis and lymph node stage. Knockdown of HNRNPA2B1 decreased the expression levels of miR-17, miR-18a, miR-20a, miR-93, and miR-106b and inhibited the proliferation of ESCA cells.

Previous studies have shown that dysregulated m6A modification is associated with a number of cancers, such as hepatocellular carcinoma, lung cancer, and pancreatic cancer (Liu G. M. et al., 2020; Liu et al., 2020a; Liu G. M. et al., 2020; Meng et al., 2020). In our data, m6A regulators played important roles in the prognosis and outcome of patients with ESCA and were closely related to tumor size. This result may indicate that m6A modification in ESCA can affect tumor growth. The results of this study are consistent with the findings of previous studies (Guo et al., 2020; Xu et al., 2020). Xu et al. (2020) observed that the expression of ALKBH5 and HNRNPC could predict the outcome of ESCA. In addition, HNRNPA2B1 is a binding protein and member of the hnRNP family (Li et al., 2020). Recently, HNRNPA2B1 was recognized as a nuclear “reader” of m6A sites and was determined to affect alternative splicing patterns (Alarcón et al., 2015). However, Wu et al. (2018) reported that m6A may not directly bind to the m6A site but may provide HNRNPA2B1 access to certain sites by the “m6A switch.” Recently, Guo et al. (2020) found that m6A levels were high in ESCC tissues and that HNRNPA2B1 could promote ESCA progression through fatty acid synthesis enzymes. Therefore, the role and mechanism of HNRNPA2B1 in m6A-mediated events warrant further study. Importantly, we elucidated the mechanism by which HNRNPA2B1 regulates the miR-17-92 cluster to affect ESCA prognosis. The results of the experimental validation were consistent with the findings of the bioinformatic analysis. Furthermore, lncRNA H19 binding to HNRNPA2B1 was able to promote colorectal cancer metastasis by inducing epithelial-to-mesenchymal transition (EMT) (Zhang et al., 2020). In head and neck cancer, HNRNPA2B1 has also been reported to contribute to EMT by regulating the splicing events of oncogenes (Gupta et al., 2020). Moreover, the loss of HNRNPA2B1 in ovarian cancer cells could inhibit malignant capability and promote apoptosis (Yang et al., 2020). In contrast, HNRNPA2B1 is a negative regulator of breast cancer metastasis (Liu et al., 2020b). These findings demonstrated that HNRNRA2B1 plays complex role in cancer progression.

It was recently demonstrated that HNRNPA2B1 mediated m6A-dependent primary microRNA processing events (Alarcón et al., 2015). Therefore, we focused on the effects of HNRNPA2B1 on microRNAs. MicroRNAs (miRNAs) are endogenous non-coding RNAs measuring 18–25 nucleotides that regulate the expression and activity of coding RNAs and play key roles in biological processes (Oliveto et al., 2017). Generally, genes for several miRNAs are organized into clusters on chromosomes, and these clusters have partially conserved sequences and are transcribed as polycistronic transcription units (Wang, 2010). The miR-17-92 cluster, also known as Oncomir-1, is one of the largest clusters closely associated with tumorigenesis (Dhawan and Scott, 2018; Bai et al., 2019). Six miRNAs from the miR-17-92 cluster and its paralogous miR-106b-25 cluster are located at 13q31.3 and 7q22.1, respectively. Moreover, dysregulation of miRNAs is implicated in various diseases, including heart disease and cancer (Kee et al., 2014; Wang et al., 2015; Yang et al., 2017). Previous studies have reported that the miR-17-92 cluster induces tumorigenesis primarily by suppressing the expression of cell cycle- and tumor suppressor-related genes (Khuu and Utheim, 2016; Gruszka and Zakrzewska, 2018). In particular, the miR-17-92 cluster was highly expressed in ESCA, and its upregulation was notably correlated with lymph node metastasis, advanced TNM stage, and poor prognosis in patients with ESCA (Chen et al., 2011). Through sequencing data, it was determined that the m6A site of A2B1 could target the miR-17-92 cluster. Notably, our results showed that after the knockdown of HNRNPA2B1, the three miRNAs from the miR-17-92 cluster and the two miRNAs from the miR-106b-25 cluster were downregulated. Moreover, previous research demonstrated that HNRNPA2B1 could load miRNAs into exosomes (Villarroya-Beltri et al., 2013). Taken together, these results helped to elucidate the role played by m6A in ESCA.

### Conclusion

The results of this study demonstrate that dysregulation of 25 m6A regulators is associated with the clinical features and outcome of patients with ESCA. HNRNPA2B1 may affect the prognosis of ESCA by regulating the miR-17-92 cluster.

### Materials and Methods

#### Data Sources

RNA-seq transcriptome, miRNA expression profiles, corresponding clinical data, and large-scale cancer patient information for 159 patients with ESCA and 10 normal esophageal tissues were obtained from TCGA datasets^1^. The RNA-seq data across all tumors and their control samples were expressed as FPKM and normalized by GDC (Genomic Data Processing) mRNA quantification analysis pipeline. The RNA expression profiles of 27 ESCA cell lines were obtained from the Cancer Cell Line Encyclopedia (CCLE) datasets^2^. We extracted the clinical data, including sex (male or female), pathological state status (stages I, II, III, and IV), TNM status, survival time and vital status (alive or dead) (Supplementary Table 1). The HNRNPA2B1-target miRNA data were obtained from the GEO database^3^. GSE70061 were selected in the present study.

#### GEO Database Verification

The datasets of ESCA patients were obtained from the GEO database (see text footnote 3). Two mRNA datasets of ESCA (GSE20347, GSE75241) were selected in the present study.

#### ESCA Patients, Sample Collection and Cell Lines

A total of 106 tumor tissues were collected from ESCC patients undergoing standard resection without chemotherapy or radiotherapy at the Cancer Hospital of Shantou University Medical College from May 2010 to August 2020 with a 10-year follow-up period. And we also collected 14 paired esophagus cancer tissues and tumor-adjacent tissues from the Cancer Hospital of Shantou University Medical College. Clinicopathological features (e.g., sex, age, TNM stage, and clinical stage) were collected to analyze the correlation with the expression of HNRNPA2B1 (Table 1). This research was approved by the Cancer Hospital of Shantou University Medical College (#2019026). All patients provided written informed consent before enrollment.

Seven human ESCA cell lines (TE1, SHEEC, KYSE-70, KYSE-140, KYSE-150, EC-109, and EC-9706) were obtained from the Oncological Research Lab in Cancer Hospital of Shantou University Medical College and were cultured in RPMI 1640 medium (Gibco, Grand Island, NY, United States) supplemented with 10% fetal bovine serum (FBS) (Gibco) with 5% CO2 at 37°C.

#### m6A Regulator Gene List Collection

We organized the 25 m6A regulators that were divided into three groups according to the function of m6A RNA regulation: “writers,” “erasers,” and “readers.” “Writers” consisted of METTL4, METTL14, METTL3, RBM15, KIAA1429, WTAP, and ZC3H13. FTO and ALKBH5 were the “erasers.” IGF2BP1/2/3, YTHDC1/2, YTHDF1/2/3, EIF3A, SRSF3/9/10, TRA2A, and HNRNPC/G/A2B1 were the “readers” and were assigned to the “translation,” “splicing,” and “decay” subgroups (Zhou et al., 2020).

#### Differential Expression and Correlation of 25 m6A Regulators

To identify differentially expressed genes in ESCA, we used the Wilcox test to identify differentially expressed genes by the “vioplot” package, with adjusted *P*-values < 0.01 and | log2 fold change| > 1 as the threshold. The correlation analysis was performed, and the results were visualized using the “corrplot” package^4^.

#### Consensus Clustering Approach

According to the mRNA expression of 25 m6A regulators, we used a consensus clustering approach to divide 123 ESCA patients into an optimal number of groups by the k-means clustering algorithm in the “ConsensusClusterPlus” R package (Wilkerson and Hayes, 2010). When the clustering index “*k*” increased 2–9, *k* = 3 had the smaller CDF value. However, *k* = 2 was eventually selected due to the selection criteria that the sample size between each group should not vary excessively. The OS difference between different clusters was calculated by the Kaplan-Meier method and log-rank test. Chi-square test was used to compare the distribution of age, gender, grade and TNM stage between two clusters.

#### m6A Prognostic Score Model for ESCA

Univariate analysis was performed to determine the prognostic value of each m6A regulator. The m6A regulators with *P* < 0.05 were reserved for subsequent analysis. LASSO regression is frequently used to build a fit generalized linear model that can achieve variable selection and regularization and be called as m6A prognostic score model. LASSO regression was performed to identify the most efficient components contributing to survival prediction by the “glmnet” package. After cross-validation, the optimal number of feature variables was determined, and the coefficient of each m6A regulator included in our m6A prognostic score model was extracted by the “coef” function. By multiplying the expression of each gene and its coefficient, the sum scores of each gene can be calculated as the risk score of each patient in the prognostic score model. The sensitivity and specificity of the model were evaluated by ROC curve and its AUC.

#### RNA Extraction and RT-qPCR

Total RNA was isolated from human ESCA cells using an RNA simple Total RNA Kit (DP419, TIANGEN, Beijing, China), and RNA was subsequently reverse-transcribed with a Mir-X^TM^ miRNA qRT-RCR TB Green Kit (638314, TAKARA, Otsu, Japan). The cDNA was used as a template for quantitative real-time PCR analysis using TB Green^@^ *Premix EX* Taq^TM^ II (RR820B, TAKARA). The relative HNRNPA2B1 expression levels were calculated using the 2^–ΔΔ*Ct*^ method with the levels normalized to β-actin mRNA. Since the endogenous U6 gene is generally used to normalize the expression of miRNAs, we calculated the expression of five miRNAs relative to U6 expression in ESCA cells. The specific primers used in this study are listed in Supplementary Table 3.

#### Immunohistochemistry

Human ESCA tumor tissue paraffin-embedded sections were deparaffinized and treated with 3% H2O2 to block the endogenous peroxidase activity for 1 h. Prior to immunohistochemistry staining, the sections were immersed in an epitope-retrieving buffer at 95–100°C for 5 min. Then the proteins in the sections were blocked by normal goat serum blocking solution at room temperature for 20 min. Subsequently, they were incubated with anti-HNRNPA2B1 antibody (1:200, sc-53531, Santa Cruz Biotechnology, Santa Cruz, CA, United States) at 4°C overnight. Next, secondary antibodies were added and incubated at 37°C for 30 min. Finally, the sections were visualized by using 3,3′-diaminobenzidine. According to the extent and intensity of staining, each specimen was assigned into four grades (negative, 1+, 2+, 3+).

#### RNA Immunoprecipitation Assay and RT-qPCR Analysis

The total RNA was isolated by TRIZOL method. Polyadenylated RNA isolated from the cells indicated were fragmented into ∼100 nt sections using the NEBNext Magnesium RNA Fragmentation Module (New England Biolabs, Ipswich, MA, United States). RNA was incubated with the m6A, HNRNPA2B1 or IgG antibody (5 μg) for immunoprecipitation overnight at 4°C, according to the standard protocol of the EpiMark N6-Methyladenosine Enrichment Kit (NEB). The enrichment of certain fragments was determined using real-time PCR. The enrichment of the RNAs was normalized to input.

#### Cell Transfection

Small interfering RNAs targeting HNRNPA2B1 were designed and synthesized. SiRNAs were added to cells following the protocol of Lipofectamine 3000 (2175582, Invitrogen, Waltham, MA, United States). Cell transfection was performed in six-well plates. Then, RNA and protein of treated cells were collected after 2–4 days. The siRNA sequences were as follows: siRNA-HNRNPA2B1: CCAUACCAUCAAUGGUCAU; siRNA-Control: GGAACAUCACCUUAGAGAU.

#### Western Blot Analysis

Total protein was extracted with RIPA buffer (Sigma, St. Louis, MO, United States) and quantified by using the BCA Protein Assay Kit (Thermo Fisher Scientific, Wilmington, DE, United States). Twenty micrograms of protein was loaded and separated by 12% SDS-PAGE. After transfer to a polyvinylidene fluoride (PVDF) membrane, the membrane was incubated overnight at 4°C with an antibody against HNRNPA2B1 (1:200, sc-53531, Santa Cruz Biotechnology) or a mouse monoclonal antibody against beta ACTIN (1:2000, ab8227, Abcam). After incubation with peroxidase-conjugated anti-mouse IgG (Santa Cruz Biotechnology) at room temperature for 1–2 h, bound proteins were visualized with ECL (34580, Thermo Fisher Scientific) and detected by using BioImaging Systems (Bio-Rad, Hercules, CA, United States). The relative protein levels were visualized by normalizing to beta-actin protein as a reference by using ImageJ.

#### Cell Proliferation Assay

Cell Counting Kit-8 (CCK-8, 96992, Sigma) was used to evaluate cell proliferation. Human ESCA cells (4 × 10^3^/well) were plated into 96-well plates for 24 h and transfected with siRNA. After incubation for 24, 48, 72, and 96 h, cells were stained with 20 μL CCK8 reagent. Absorbance was measured at 450 nm wavelength by using a microplate reader to calculate the relative cell proliferation rate.

#### Statistical Analysis

Statistical analyses were performed using R (version 4.0.2)^5^. Continuous variables are expressed as the mean and standard error of the mean and were compared using Student’s *t* tests or Wilcoxon tests. Categorical variables were compared using the chi-square test. Correlation analysis was carried out using the Spearman method with the “cor.test” function. Survival analysis was performed using the Kaplan–Meier method and compared with log-rank tests using the “survival” and “survminer” packages. Multivariate Cox regression models were trained using the “coxph” function in the “survival” package. *P* < 0.05 was considered to be significant for all tests.

## Data Availability Statement

The datasets presented in this study can be found in online repositories. The names of the repository/repositories and accession number(s) can be found in the article/Supplementary Material.

## Ethics Statement

The studies involving human participants were reviewed and approved by The Cancer Hospital of Shantou University Medical College. The patients/participants provided their written informed consent to participate in this study.

## Author Contributions

WC, KL, JC, and DZ conceived and designed the project, acquired research data, and wrote the manuscript. KL, XL, YL, BQ, DX, SM, and YW acquired research data. JC provided clinical tissue sections and cell lines. WC and DZ contributed to helpful discussion and reviewed the manuscript. All authors read and approved the final manuscript.

## Conflict of Interest

The authors declare that the research was conducted in the absence of any commercial or financial relationships that could be construed as a potential conflict of interest.

## Footnotes

1 https://cancergenome.nih.gov/

2 https://portals.broadinstitute.org/ccle/about

3 http://www.ncbi.nlm.nih.gov/geo/

4 https://github.com/taiyun/corrplot

5 www.r-project.org
