# Supplementary material for: HNRNPA2B1 Affects the Prognosis of Esophageal Cancer by Regulating the miR-17-92 Cluster
Source: Front Cell Dev Biol. 2021 Jun 30;9:658642. doi: 10.3389/fcell.2021.658642 (PMC8278577; doi:10.3389/fcell.2021.658642)
Supplement: Supplementary file 1 [file Data_Sheet_1.docx]

Supplementary Material

# Supplementary Figures and Tables

## Supplementary Figures


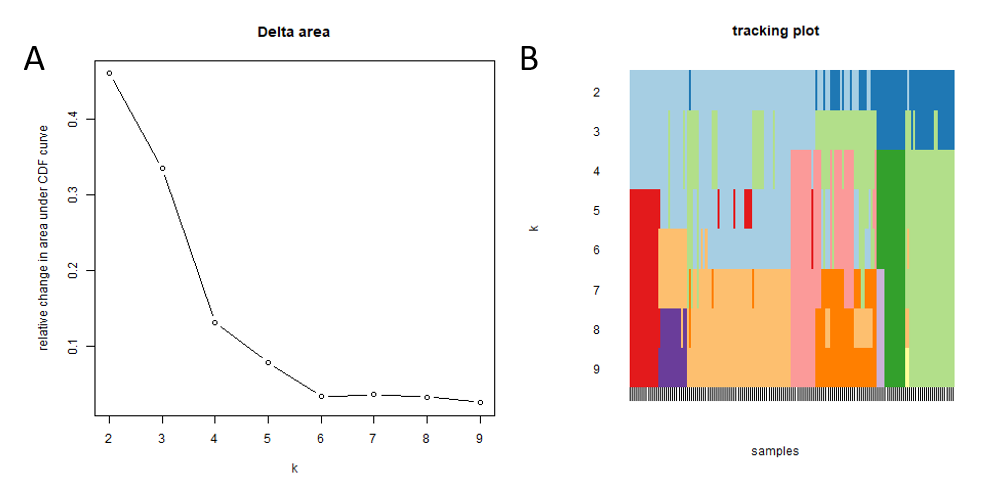


### Supplementary Figure 1. Consensus clustering identified two clusters of patients with ESCA. (A) Area under CDF curve when *k* ranges from 2 to 9. (B) Distribution of each sample when *k* ranges from 2 to 10.

**
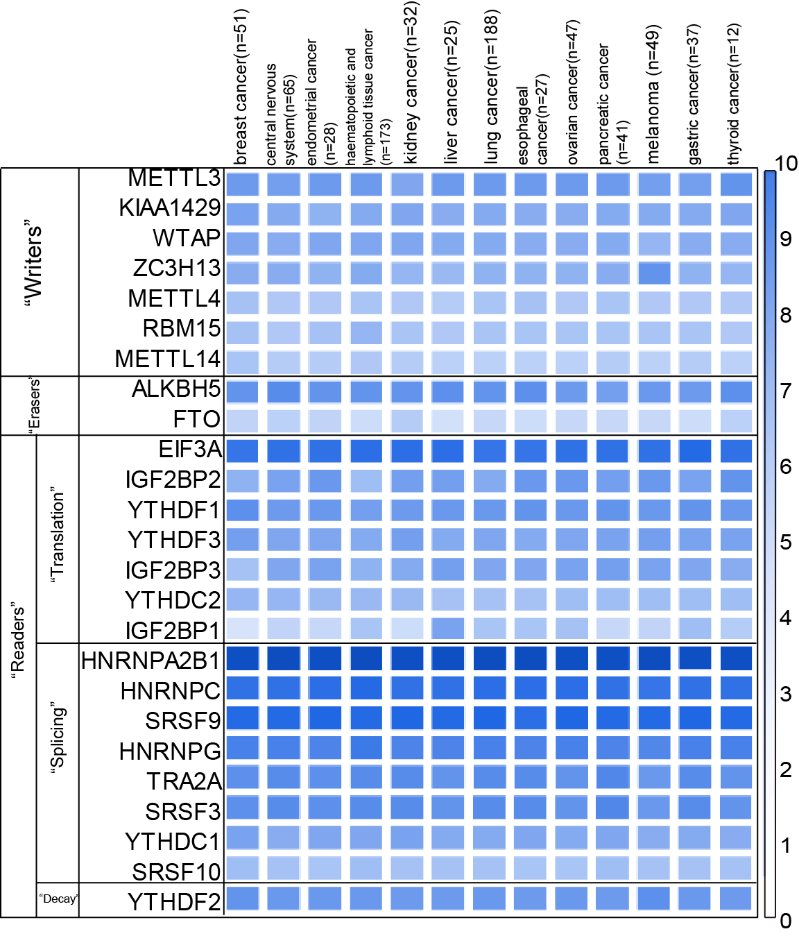
**

### Supplementary Figure 2. The expression level of 25 m6A regulators in 13 types of cancer cell lines from the CCLE database. The gene expression of log-scale was used by log^2^(RKPM + 1).

**
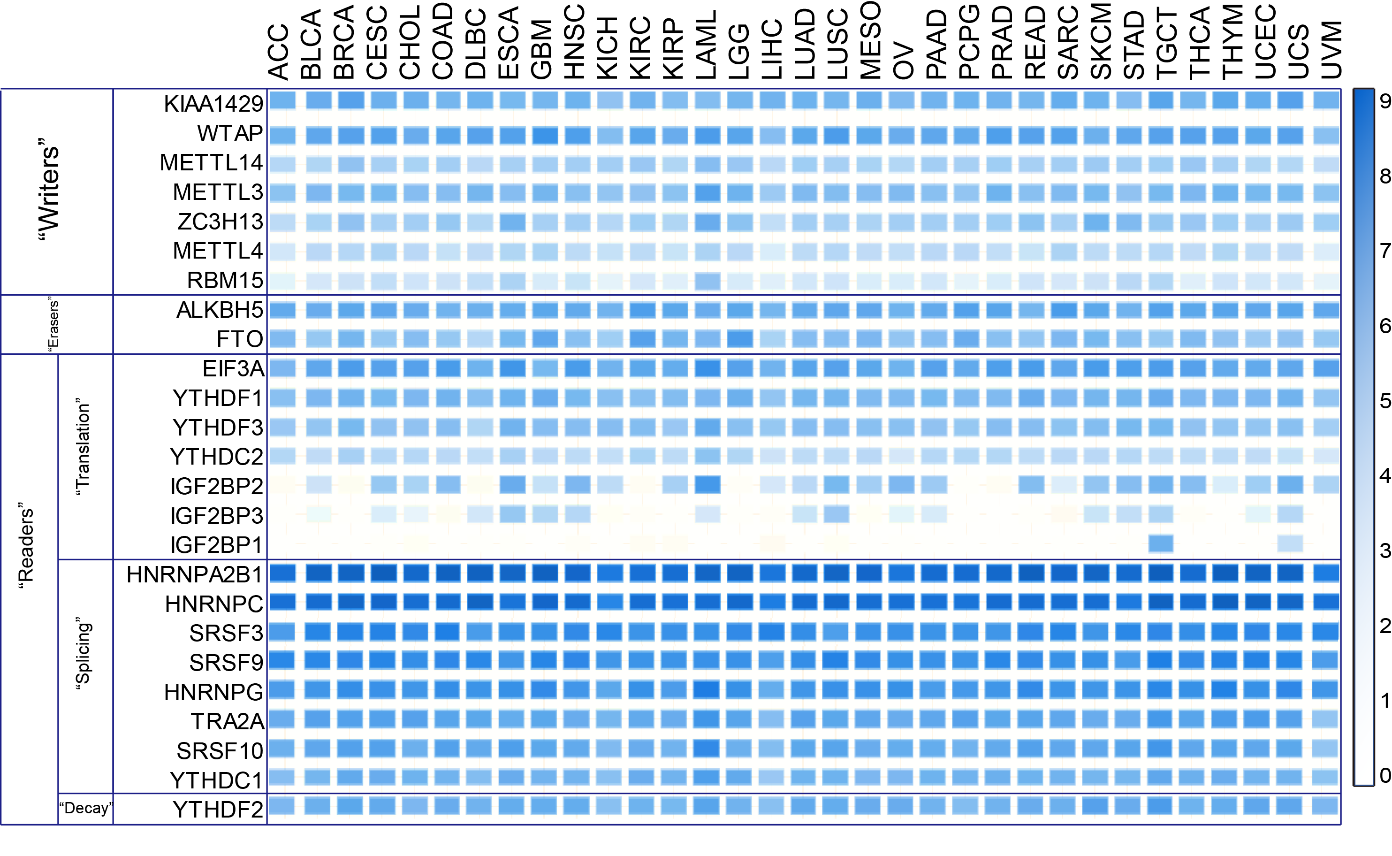
**

### Supplementary Figure 3. The expression level of 25 m6A regulators in the 33 types of cancer from TCGA datasets. The gene expression of log-scale was used by log^2^(TPM + 1).

## Supplementary Tables

### Supplementary Table 1. The general clinical information of patients with ESCA in TCGA.

| Variables | | Cluster | | P value |
| --- | --- | --- | --- | --- |
|  |  | EC1 | EC2 |  |
| Fustat | Alive | 53 | 26 | **<0.001** |
|  | Dead | 28 | 16 |  |
|  |  |  |  |  |
| Gender | FEMALE | 11 | 8 | 0.086 |
|  | MALE | 70 | 34 |  |
|  |  |  |  |  |
| Stage | Stage I | 10 | 2 | **<0.001** |
|  | Stage II | 42 | 21 |  |
|  | Stage III | 27 | 14 |  |
|  | Stage IV | 2 | 5 |  |
|  |  |  |  |  |
| Tumor Stage | T1 | 15 | 8 | 0.710 |
|  | T2 | 25 | 10 |  |
|  | T3 | 38 | 24 |  |
|  | T4 | 3 | 0 |  |
|  |  |  |  |  |
| Distant metastasis | M0 | 79 | 37 | **<0.001** |
|  | M1 | 2 | 5 |  |
|  |  |  |  |  |
| Nodal Stage | N0 | 41 | 16 | 0.014 |
|  | N1 | 31 | 24 |  |
|  | N2 | 6 | 2 |  |
|  | N3 | 3 | 0 |  |
|  |  | n=81 | n=42 |  |

### Supplementary Table 2. The target genes for miRNAs from DIANA tools.

| **Pathway** | **Target genes** | | | | |
| --- | --- | --- | --- | --- | --- |
|  | **miR-17** | **miR-106b** | **miR-93** | **miR-18a** | **miR-20a** |
| TGF-beta | ROCK2 | SMAD6,PPP2CA,SMURF2,ROCK2,SMAD4,E2F5,SMURF1,ZFYVE9,RBL1,SMAD5,BMP2,MAPK1,TGFBR2,BMPR2 | ROCK2 | THBS1 | SMAD6,PPP2CA,SMURF2,ROCK2,SMAD4,E2F5,ZFYVE9,RBL1,SMAD5,BMP2,EP300,MAPK1,TGFBR2,BMPR2 |
| Wnt | TBL1X,CCND2,ROCK2,PPP3CA | FZD7,TCF4,VANGL1,PPP2CA,ROCK2,TCF7L1,NFATC4,FZD4,NFAT5,CCND1,SMAD4,MAPK8,CHP2 | TBL1X,CCND2,ROCK2,PPP3CA | DAAM2,CCND2,PRKACB | FZD7,TCF4,VANGL1,PPP2CA,CCND2,ROCK2,TCF7L1,NFATC4,FZD4,NFAT5,MAPK9,CCND1,SMAD4,MAPK8,EP300,CHP2 |
| RNA degradation | CNOT7,RQCD1 | CNOT6,CNOT4,BTG2,CNOT7,XRN1,BTG3,CNOT6L | CNOT7,RQCD1 | DCP2,BTG3 | CNOT6,CNOT4,BTG2,CNOT7,XRN1,BTG3,CNOT6L |
| Cell cycle | CCND2,CDKN1A | RBL2,E2F1,WEE1,CCND1,SMAD4,E2F5,E2F3,RBL1,CDC23,CDKN1A | CCND2,CDKN1A | ORC2 | RBL2,E2F1,CCND2,WEE1,CCND1,SMAD4,E2F5,E2F3,RBL1,CDC23,EP300,CDKN1A |
| p53 | CCND2,CDKN1A,SESN3 | CCND1,TP73,TNFRSF10B,CDKN1A,RRM2,SESN3,CCNG2 | CCND2,CDKN1A,SESN3 | THBS1,CCND2,ATM | CCND2,CCND1,TP73,CDKN1A,RRM2,SESN3,CCNG2 |
| MAPK | NTRK2,CRK,MAP3K1,TAOK1,PPP3CA,SOS1,MKNK2,MAP3K5 | TAOK3,NTRK2,PDGFRA,DUSP2,RASA2,ELK4,CRK,MAPK7,RASGRF2,MAP3K1,RRAS2,TAOK1,NFATC4,DUSP10,RASA1,MAPK8,SOS1,NF1,MAP3K2,MKNK2,MAPK1,CHP2,TGFBR2,MAP3K5 | NTRK2,CRK,MAP3K1,TAOK1,PPP3CA,SOS1,MKNK2,MAP3K5, | MAP3K1,TAOK1,CDC42,FGF1,PRKACB | TAOK3,NTRK2,PDGFRA,DUSP2,RASA2,ELK4,CRK,MAPK7,RASGRF2,MAP3K1,RRAS2,TAOK1,NFATC4,DUSP10,RASA1,MAPK9,MAPK8,SOS1,NF1,MAP3K2,MKNK2,MAPK1,CHP2,TGFBR2,MAP3K5 |
| mTOR | VEGFA,PTEN | VEGFA | STK11,VEGFA | HIF1A,PTEN | HIF1A,VEGFA,PTEN |

### Supplementary Table 3. The sequences of the quantitative real-time polymerase chain reaction primers.

| RT-PCR | Primer sequence (5'-3') |
| --- | --- |
| HNRNPA2B1 | Forward: CAGTTCTCACTACAGCGCCA |
|  | Reverse: TTCCTCTCCAAAGGAACAGTTT |
| ACTIN | Forward: GTCTGCCTTGGTAGTGGATAATG |
|  | Reverse: TCGAGGACGCCCTATCATGG |
| U6 | Forward: CTCGCTTCGGCAGCACA |
|  | Reverse: AACGCTTCACGAATTTGCGT |
| miRNAs Universal reverse | GTGCAGGGTCCGAGGT |
| miR-17 | Forward: CAAAGTGCTTACAGTGCAGGTAG |
| miR-18a | Forward: TAAGGTGCATCTAGTGCAGATAG |
| miR-20a | Forward: TAAAGTGCTTATAGTGCAGGTAG |
| miR-93 | Forward: CAAAGTGCTGTTCGTGCAGGTAG |
| miR-106 | Forward: TAAAGTGCTGACAGTGCAGAT |
